# Evaluating the NIRS-derived microvascular O_2_ extraction “reserve” in groups varying in sex and training status using leg blood flow occlusions

**DOI:** 10.1371/journal.pone.0220192

**Published:** 2019-07-25

**Authors:** Erin Calaine Inglis, Danilo Iannetta, Juan M. Murias

**Affiliations:** Faculty of Kinesiology, University of Calgary, Calgary, AB, Canada; São Paulo State University (UNESP), BRAZIL

## Abstract

It has been demonstrated that the plateau in the near-infrared spectroscopy (NIRS) derived deoxygenated hemoglobin and myoglobin (deoxy[Hb+Mb]) signal (i.e., deoxy[Hb+Mb]_PLATEAU_) towards the end of a ramp-incremental (RI) test does not represent the upper-limit in O_2_ extraction of the vastus lateralis (VL) muscle, given that an O_2_ extraction reserve has been recently observed. This study aimed to investigate whether this O_2_ extraction reserve was present in various populations and whether it exhibited sex- and/or training- related differences.Sixteen men- 8 untrained (27±5 years; 83±11 kg; 179±9 cm), 8 trained (27±4 years; 82±10 kg; 182±8 cm) and 9 trained women (27±2 years; 66±10 kg; 172±6 cm) performed a RI cycling test to exhaustion. The NIRS-derived deoxy[Hb+Mb] signal was measured continuously on the VL as a proxy for O_2_ extraction. A leg blood flow occlusion (i.e., ischemia) was performed at rest (LBF_OCC_ 1) and immediately post the RI test (LBF_OCC_ 2).No significant difference was found between the deoxy[Hb+Mb] amplitude during LBF_OCC_ 1 and the deoxy[Hb+Mb]_PLATEAU_ (*p*>0.05) nor between baseline (bsln) deoxy[Hb+Mb] values. deoxy[Hb+Mb] amplitude during LBF_OCC_ 2 was significantly greater than LBF_OCC_ 1 and at deoxy[Hb+Mb]_PLATEAU_ (*p*<0.05) with group means ~30–45% higher than the deoxy[Hb+Mb]_PLATEAU_ and LBF_OCC_ 1 (*p*<0.05). No significant differences were found between groups in O_2_ extraction reserve, regardless of sex- or training-statusThe results of this study demonstrated the existence of an O_2_ extraction reserve in different populations, and that neither sex- nor training-related differences affect the amplitude of the reserve.

## Introduction

The examination of muscle metabolism has revealed the presence of a functional reserve in potential energy at the limit of exercise tolerance [1,2]. Recently, using transient ischemia (i.e., leg blood flow occlusion), the existence of an O_2_ extraction “reserve” (as measured by the near-infrared spectroscopy (NIRS) derived deoxygenated [hemoglobin and myoglobin] (deoxy[Hb+Mb]) signal) was found in the superficial vastus lateralis (VL) muscle as well as in other areas and depths of the quadriceps muscles at the end of ramp-incremental exercise [3,4]. Similarly, previous studies evaluating the deoxy[Hb+Mb] signal have performed occlusions either before ramp-exercise [5,6] or a few minutes after its cessation [7–10]. These studies also demonstrated that O_2_ extraction does not reach its upper limit during exercise at $\dot{V}$O_2peak_ when compared to the ischemic condition imposed by the occlusion. This is particularly relevant in regards to our previous study in which an occlusion performed immediately upon exercise cessation revealed that the deoxy[Hb+Mb] signal could still be increased despite a change in slope (i.e., the deoxy[Hb+Mb] break point (deoxy[Hb+Mb]_BP_)) and subsequent plateau (deoxy[Hb+Mb]_PLATEAU_) that is typically seen towards the end of the test (e.g., >80% $\dot{V}$O_2peak_). These studies also suggested that, assuming the continuous increase in local muscle $\dot{V}$O_2_ [11], the attenuation of the deoxy[Hb+Mb] signal may indicate that there is a greater perfusion of blood in the area being investigated, as blood flow to the legs has also been shown to continually increase during incremental exercise [11]. In this context, greater blood flow perfusion triggered by an increased concentration of local vasodilatory substances associated with exercising in the severe intensity domain [12], is what could support the increasing metabolic rate [13]. Given the homogeneity of the groups investigated in these studies and a lack of group comparisons [3,4], it is currently unknown whether this O_2_ extraction reserve measured immediately at the end of ramp-exercise is present and/or different in magnitude in other populations which might present different O_2_ extraction capacities.

Differences in O_2_ extraction capacity have been related to different levels of fitness. While, it has been shown that during ramp-incremental exercise, trained cyclists demonstrated a right-shift in the deoxy[Hb+Mb] pattern compared to physically active individuals [14], it has been demonstrated that training increases capillary-to-muscle fiber ratio [15,16] and capillary permeability [17], as well as mitochondria content and function [18,19]; all of these key adaptations leading to a greater systemic arteriovenous O_2_ difference [20] and to a greater amplitude of the deoxy[Hb+Mb] signal (a proxy for local maximal O_2_ extraction capacity) in trained individuals compared to untrained [21]. Moreover, sex-related differences in hemodynamic responses to exercise have been reported to lead to variations in the rate of local fractional O_2_ extraction during ramp-incremental exercise [22]. Specifically, a previous study [22] indicated that when exercising at intensities above ~50% of $\dot{V}$O_2peak_ during RI exercise, women relied more on fractional O_2_ extraction for a given relative increase in metabolic rate, possibly due to poorer matching between O_2_ delivery and utilization and/or limitations resulting from lower hemoglobin carrying capacity compared to men [23,24]. Additionally, it has been suggested that there are sex-related differences in leg vasodilatory capacity, as women demonstrated a greater hyperemic responses to exercise, greater exercise-induced femoral dilation, and augmented femoral vascular conductance to age-matched men [25].

Thus, we hypothesized that these structural and functional differences in vascular and metabolic dynamics that determine and/or affect O_2_ extraction capacity could impact the magnitude of the O_2_ extraction reserve immediately at the end of ramp-incremental exercise. Specifically, given the greater oxidative capacity of trained individuals as well as the larger reliance on fractional O_2_ extraction of women, these groups could have a greater ability to sustain a higher O_2_ flux, leading to a reduced magnitude of O_2_ extraction reserve. Therefore, the aim of the present study was to investigate whether the O_2_ extraction reserve, identified as an overshoot in the deoxy[Hb+Mb] signal immediately at the end of a RI test to exhaustion, would be present and different in magnitude in three groups differing for sex (trained men vs trained women) and training status (trained vs untrained men).

## Methods

### Participants

25 participants [16 healthy men—8 untrained (27 ± 5 years; 83 ± 11 kg; 179 ± 9 cm), 8 trained (27 ± 4 years; 82 ± 10 kg; 182 ± 8 cm) and 9 healthy trained women (27 ± 2 years; 66 ± 10 kg; 172 ± 6 cm)] volunteered and gave their written consent to participate in this study after completing the physical activity readiness questionnaire (PARQ+) and being cleared for exercise. Participants ranged from untrained (not engaging in any structured training regimen) to trained (ranging from recreationally trained individuals who regularly engage in aerobic training programs 3–4 times per week for 1–2 hours per session to amateur cyclists training 5–6 days per week for 1.5–4 hours per session). Data from 11 participants that have been presented elsewhere for the identification of the O_2_ extraction reserve [3] were included in this larger dataset. All participants were non-smokers, non-obese, with no cardiovascular disease and were not undergoing any medical treatment that could potentially affect their cardiopulmonary and metabolic responses to exercise. The Conjoint Health Research Ethics Board at the University of Calgary approved all procedures included in this study.

### Protocol

Each participant required a single visit to the laboratory which included two leg blood flow occlusions (LBF_OCC_)- before and immediately after a RI test to exhaustion that was performed on an electromagnetically braked cycle ergometer (Velotron Dynafit Pro, Racer Mate, Seattle, WA, USA). All participants were familiar with performing maximal efforts and the majority of them had previously performed maximal testing in our laboratory.

The RI test consisted of a 4-min warm-up at 50 W followed by a 30 W·min^-1^ (1 W every 2 s) and 25 W·min^-1^ (1 W every 2.4 s) ramp for men and women respectively. Throughout the test the participants cycled at their preferred cadence (between ~80–90 rpm) and were asked to maintain this cadence (± 3 rpm) until they reached the limit of their exercise tolerance. The RI test was stopped when the cadence dropped by more than 15 RPM or when volitional exhaustion occurred despite strong verbal encouragement.

A LBF_OCC_ was performed using an automatic rapid (0.3 s) cuff inflation system (Hokanson, Inc., Bellevue, USA). The cuff (13 x 85 cm) was placed on the uppermost portion of the thigh of the right leg. The protocol began with a resting period for baseline (bsln 1) measurements of deoxy[Hb+Mb] (4-min) where participants were seated on the bike with their feet secured on the pedals. The pedals were positioned at mid-rotation (equal height) with the right pedal forward and supported by a block to avoid movement and ensure a relaxed position. The first occlusion (LBF_OCC_ 1) consisted of an 8-min period during which the cuff was inflated to a pressure of 300 mmHg. A pressure of 300 mmHg was used for all participants during all occlusions to ensure standardization between participants as well as to account for increases in arterial pressure from rest to exercise. From the end of LBF_OCC_ 1 to the start of the RI test, a 20-min recovery period was provided to allow for the conditions of the tissues to return to baseline. This recovery period included 8-min of NIRS measurement without movement followed by 12-min of final preparation for the ramp-incremental test (i.e., to apply the mask and make any final adjustments). The second occlusion (LBF_OCC_ 2) was performed immediately after the end of the RI exercise for 2-min. The researchers placed the right leg in the identical position of that used during LBF_OCC_ 1 and used the same occlusion pressure as LBF_OCC_ 1. The aim of each occlusion was to achieve the highest level of O_2_ extraction for the given metabolic state.

### Measurements

NIRS-derived deoxy[Hb+Mb] and total [hemoglobin + myoglobin] (total[Hb+Mb]) were measured continuously in the VL muscle of the right leg by means of NIRS (Oxiplex TS; ISS, Champaign, USA) at a sampling rate of 2 Hz throughout the entire protocol. The source detector differences were 2.0, 2.5, 3.0, 3.5 cm. Further details on the specifics of this system can be found elsewhere [3,26]. The NIRS probe was placed on the belly of the VL muscle midway between the inguinal crease and the proximal border of the patella and was secured in place by double-sided tape and an elastic strap to prevent any movement. The probe was covered by an optically dense, black vinyl sheet and an elastic bandage to minimize both the intrusion of external light and movement of the probe. A Harpenden skinfold caliper was used to measure skin and adipose tissue thickness at the area of the NIRS probe interrogation on the VL.

A breath-by-breath metabolic cart (Quark CPET, Cosmed, Rome, Italy) was used to measure gas-exchange variables and pulmonary ventilation (inspired and expired flow rates) starting at the beginning of the pre-RI test baseline at which point it was synchronized with the NIRS measurement. Expired gases were sampled at the mouth and analyzed for fractional concentrations of O_2_ and CO_2_ after calibration with precision-analyzed gas mixtures, according to manufacturer specifications. The flowmeter was calibrated using a syringe of known volume (3 liters).

### Data and analysis

#### Deoxy[Hb+Mb] signal

To determine the deoxy[Hb+Mb] signal at the time points of interest, bin-averaging strategy was used as follows in accordance with a previous study [3]: bsln 1: 45s average starting at minute 3; bsln 2: 45s average starting at minute 7 after cuff release post LBF_OCC_ 1; LBF_OCC_ 1: highest 10s average during the last 2 minutes of the occlusion; deoxy[Hb+Mb]_PLATEAU_: highest 10s average from the deoxy[Hb+Mb]_BP_ until the RI test termination; LBF_OCC_ 2: highest 10s average during the entire occlusion. Normalization for amplitude of the deoxy[Hb+Mb] signal was done on an individual basis with the amplitude from bsln 2 to the deoxy[Hb+Mb]_PLATEAU_ representing 100% amplitude, as previous work has found that the deoxy[Hb+Mb]_PLATEAU_ and resting baseline occlusions to not be statistically different [3] and no differences were found between baseline 1 and 2. Additionally, to compare slopes between groups for the rate of increase in the deoxy[Hb+Mb] signal throughout the RI test, data were normalized from 0 to 100% of the peak power output (PO), as previously suggested [22].

As previously described [27], the deoxy[Hb+Mb]–time relationship related to the ramp portion of the RI test was modeled with the following piece-wise “double-linear” model:

f=if(x<BP,g(x),h(x))

$$g\left(\text{x}\right)\;=i_{1}+\;\left(s_{1}\cdot \;\text{x}\right)$$

$$i_{2}=i_{1}+\;\left(s_{1}\cdot BP\right)$$

$$h\left(\text{x}\right)\;=i_{2}+\;\left(s_{2}\cdot \;\left(\text{x}—BP\right)\right)$$

fitftoy,

where *f* is the double-linear function, x is time and y is deoxy[Hb+Mb], *BP* is the time coordinate corresponding to the interception of the two regression lines (i.e., the deoxy[Hb+Mb]_BP_), *i*_1_ and *i*_2_ are the intercepts of the first and second linear function respectively and *s*_1_ and *s*_2_ are the slopes. Model parameter estimates for each participant were determined by linear least-square regression analysis. Data that were ± 3 SD from the local mean were removed. The double linear fit started at the onset of the systematic increase in the deoxy[Hb+Mb] signal until the last data point corresponding to the end of the test. Specifically, the deoxy[Hb+Mb]_BP_ was detected as the point at which the model fit would detect a significant attenuation in the slope of the increase.

#### Adipose tissue thickness correction

A correction factor based on the relationship of adipose tissue thickness and total[Hb+Mb] was utilized, in order to account for the influence of subcutaneous adipose tissue, as previously described [28]. Briefly, a linear regression was calculated based on the skinfold thickness under the probe interrogation site and the total[Hb+Mb] signal (2 min average during bsln 1). Using the regression equation, the difference (total[Hb+Mb]_diff_) between the actual recorded value (total[Hb+Mb]) and the expected value (skinfold value substituted as ‘x’ into the linear equation) was calculated. An individual correction factor was then derived as follows: *Individual Correction Factor* = (y-intercept (x = 0) + total[Hb+Mb]_diff_) / total[Hb+Mb]. Each individual correction factor was then applied to the deoxy[Hb+Mb] value at the selected time points.

#### Gas exchange parameters

Breath-by-breath $\dot{V}$O_2_ data were individually analyzed: irregular data points that were ± 3 SD from the local mean were removed before the data were linearly interpolated to 1 s intervals. Subsequently, the second-by-second data were time aligned so that the onset of the RI test represented time “zero”. In order to account for the lag time in the increase in $\dot{V}$O_2_ after the onset of the ramp portion, the mean response time was calculated (Origin, Origin Lab, Northampton, USA) on an individual basis as previously described [29]. Briefly, a “double linear model” was fit from bsln to the previously established gas exchange threshold (GET). The mean response time corresponded to the time delay between the onset of the RI test (i.e., 240 s of baseline) and the intersection of the forward extrapolation of the bsln $\dot{V}$O_2_ (slope constrained to “zero”) and backwards extrapolation of the linear $\dot{V}$O_2_-time relationship from GET.

In order to determine RCP and GET, two exercise physiologists independently performed a visual inspection of the ventilatory and gas exchange profiles, as previously described [30]. Briefly, the RCP corresponded to the second disproportional increase (second breakpoint) in the $\dot{V}$_E_/$\dot{V}$O_2_ relationship, where the end-tidal PCO_2_ began to fall after a period of isocapnic buffering. For confirmation of the RCP the relationship between $\dot{V}$_E_/$\dot{V}$CO_2_ against $\dot{V}$O_2_ was also considered. GET corresponded to the point at which $\dot{V}$CO_2_ began to increase out of proportion in relation to $\dot{V}$O_2_, coinciding with a systemic rise in the $\dot{V}$_E_-to-$\dot{V}$O_2_ relationship and end-tidal PO_2_ where the ventilatory equivalent of $\dot{V}$CO_2_ ($\dot{V}$E/$\dot{V}$CO_2_) and end-tidal PCO_2_ are stable. If there was a disagreement of more than 100 mL∙min^-1^ in the results between the physiologists, a second conjoint evaluation was performed until a consensus was reached. $\dot{V}$O_2peak_ was defined as the highest $\dot{V}$O_2_ computed from a 30s rolling average. Peak PO was the highest power output value obtained at the end of the RI test.

### Statistics

All statistics were performed using SPSS version 23 (SPSS, Chicago, USA) with statistical significance set at a *P* < 0.05. Descriptive data are presented as mean ± SD. Where appropriate a LSD post-hoc test was applied. A repeated-measure ANOVA (3 groups x 5 times points) was used to detect differences in the deoxy[Hb+Mb] data at the selected time points (bsln 1, LBF_OCC_ 1, bsln 2, deoxy[Hb+Mb]_PLATEAU_, and LBF_OCC_ 2). A one-way ANOVA test was used to detect group differences in variables derived from the RI test (i.e., peak power output, $\dot{V}$O_2peak_, RCP, the deoxy[Hb+Mb]_BP_, and blood lactate concentration ([BLa])), as well as RI slopes. To compare the $\dot{V}$O_2_ associated with the RCP and deoxy[Hb+Mb]_BP_, a paired t-test was used. To quantify the relationship between NIRS variables and $\dot{V}$O_2peak_, a Pearson product moment correlation was calculated.

## Results

### Anthropometrics

Women displayed a larger mean adipose tissue thickness (14.2 ± 3.9 mm) compared to both trained (6.0 ± 1.4 mm; *p* < 0.05) and untrained men (7.1 ± 1.8 mm; *p* < 0.05). Adipose tissue thickness was not different between trained and untrained men (*p* > 0.05).

### Ramp-incremental test

Results from the RI test can be found in Table 1. A difference in absolute $\dot{V}$O_2peak_ and peak PO was found between all groups (*p* < 0.05), whereas relative $\dot{V}$O_2peak_ was greater in trained men compared to untrained men (*p* < 0.05) with no difference found between trained women and trained men (*p* > 0.05). The absolute $\dot{V}$O_2_ associated with the RCP was different in all groups, with trained men displaying the highest value, followed by untrained men and trained women (*p* < 0.05; Table 1). No differences were found between groups in RCP expressed as a percent of $\dot{V}$O_2peak_ (*p* > 0.05)_._ The absolute $\dot{V}$O_2_ associated with the deoxy[Hb+Mb]_BP_ was greater in the trained men compared to the untrained men as well as trained women (*p* < 0.05), with no between group differences found in the percent $\dot{V}$O_2peak_ associated with the deoxy[Hb+Mb]_BP_ (*p* > 0.05). Additionally, in all groups the $\dot{V}$O_2_ associated with the RCP and the deoxy[Hb+Mb]_BP_ were not different (*p* > 0.05).

**Table 1:** Ramp incremental test results including peak power output (Peak PO), peak rate of oxygen uptake ($\dot{V}$O_2peak_), respiratory compensation point (RCP), and the deoxygenated [hemoglobin + break point] (deoxy[Hb+Mb]_BP_).

		Peak PO	$\dot{V}$O_2peak_		RCP		deoxy[Hb+Mb]_BP_	
		(W)	(L∙min^-1^)	(mL∙kg∙min^-1^)	(L∙min^-1^)	% $\dot{V}$O_2peak_	(L∙min^-1^)	% $\dot{V}$O_2peak_
Men	Trained	433±34	4.70±0.53	57.6±5.2	3.94±0.39	82.7±7.3	3.99±0.69	83.6±9.8
	Untrained	348±27^a^	3.81±0.36^a^	46.6±6.0^a^	3.21±0.22^a^	84.5±4.5	3.40±0.50^a^	88.9±9.0
Women	Trained	326±26^a^	3.34±0.41^a^^,^^b^	51.3±7.2	2.85±0.32^a^^,^^b^	85.5±3.3	2.96±0.38^a^	88.6±9.0

^a^, significantly different from trained men;

^b^, significantly different from untrained men. Statistical significance set at *p* < 0.05.

### Deoxy[Hb+Mb] amplitude during occlusions and ramp-incremental exercise

**Fig 1:**
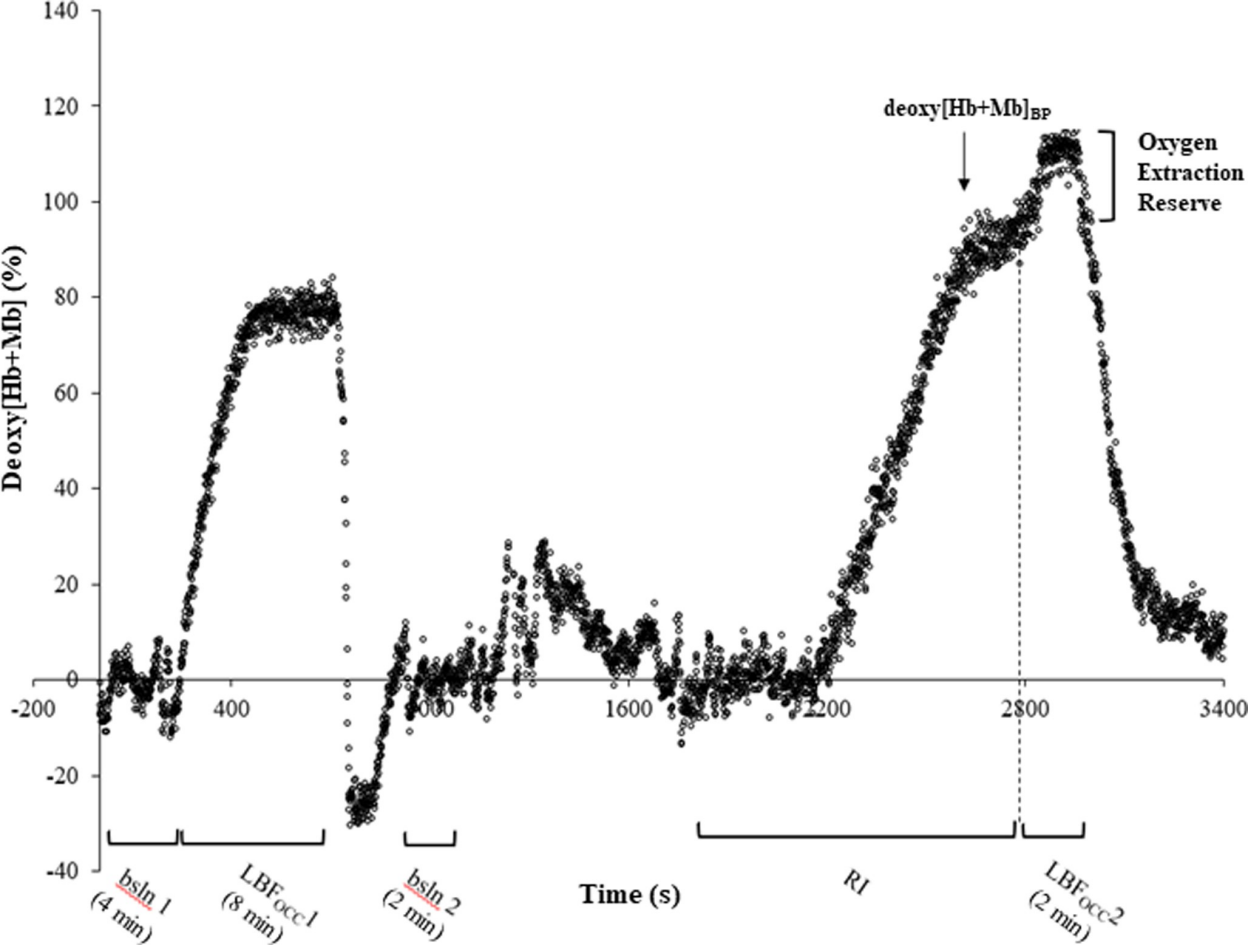
Overview of the deoxygenated hemoglobin concentration (deoxy[Hb+Mb]) signal during the entire protocol for one participant (bsln 1 and 2, Baseline 1 and 2; LBF_OCC_ 1 and 2, leg blood flow occlusion 1 and 2; RI, ramp incremental test; deoxy[Hb+Mb]_BP_, deoxygenated hemoglobin concentration break point).

See Fig 1 for an overview of the deoxy[Hb+Mb] signal during the entire protocol for one participant. Absolute values for the amplitude of the deoxy[Hb+Mb] data are presented in Table 2. Within each group no differences were found between deoxy[Hb+Mb] values measured at baseline 1 and baseline 2 (*p* > 0.05). In all three groups the deoxy[Hb+Mb] amplitude during LBF_OCC_ 2 was greater than during both LBF_OCC_ 1 and the ramp-incremental test (*p* < 0.05). The deoxy[Hb+Mb] amplitude measured during LBF_OCC_ 1 and at the deoxy[Hb+Mb]_PLATEAU_ were not different (*p* > 0.05). Normalized deoxy[Hb+Mb] amplitude data produced the same statistical findings as the absolute deoxy[Hb+Mb] amplitude data. No sex- or training-related differences were found within the delta deoxy[Hb+Mb] values *(p* > 0.05). At each time point the amplitude of the absolute deoxy[Hb+Mb] was greater in trained men when compared to women (*p* < 0.05). No difference was found between the trained and untrained men nor between women and untrained men with regards to the deoxy[Hb+Mb] amplitude (*p* > 0.05).

**Table 2:** Amplitude of the deoxygenated hemoglobin (deoxy[Hb+Mb]) values during the leg blood flow occlusions (LBF_OCC_ 1 and 2) and at the deoxy[Hb+Mb] plateau (deoxy[Hb+Mb]_PLATEAU_) for all groups. Normalized values are expressed as a percentage relative to the deoxy[Hb+Mb] amplitude from baseline 1 to the deoxy[Hb+Mb]_PLATEAU_.

			LBF_OCC_ 1	deoxy[Hb+Mb]_plateau_	LBF_OCC_ 2
Absolute (μmol∙L^-1^)	Men	Trained	21.0±7.8†	20.4±9.1†	26.6±11.3#†
		Untrained	14.6±9.5	14.6±8.5	18.9±9.1#
	Women	Trained	9.7±3.5	9.8±3.6	14.0±5.3#
Normalized (%)	Men	Trained	106.5±15.9	100.0±0.0	133.7±13.7#
		Untrained	99.1±19.1	100.0±0.0	137.7±29.4#
	Women	Trained	103.8±32.8	100.0±0.0	144.5±29.5#

#, significantly different all other time points;

†, significantly different than women.

Statistical significance set at *p* < 0.05.

### Deoxy[Hb+Mb], total[Hb+Mb] and saturation

**Fig 2:**
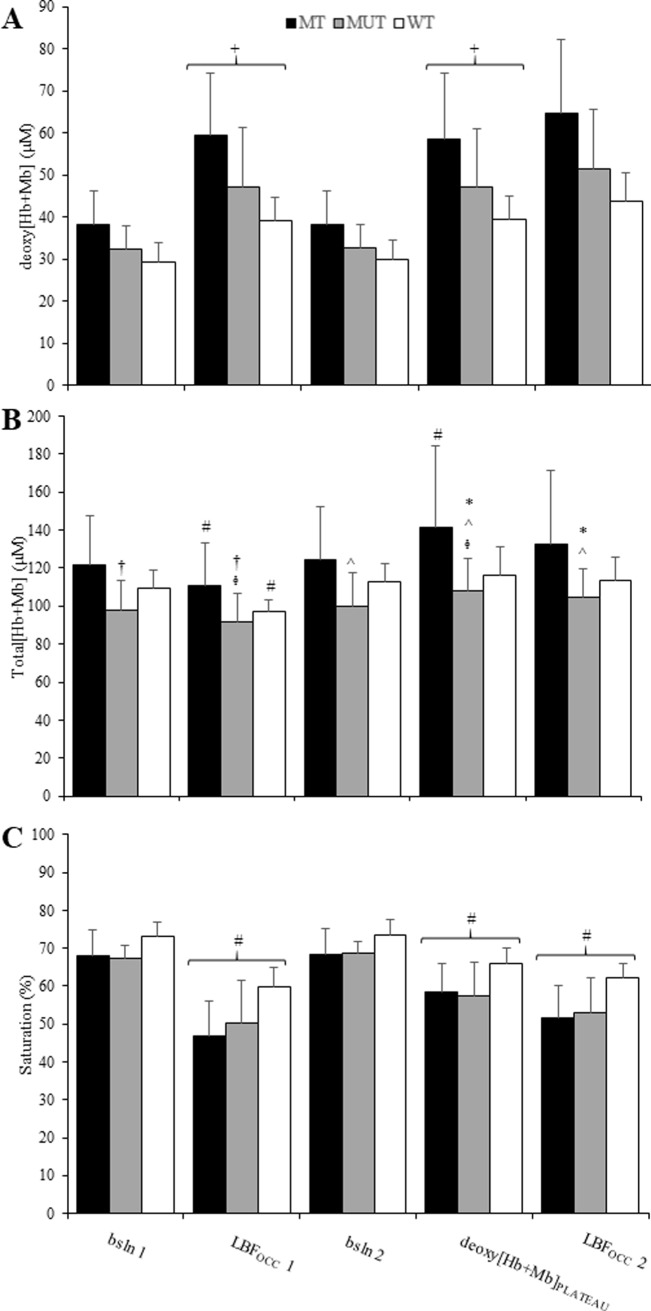
NIRS-derived measurements at different time points throughout the protocol for trained men (MT), untrained men (MUT) and trained women (WT). *PANEL A*: deoxyhemoglobin concentration, (deoxy[Hb+Mb]), at all points trained men significantly different than women; *PANEL B*: Total [hemoglobin + myoglobin] (total[Hb+Mb]), at all points trained men significantly different than untrained men; *PANEL C*: tissue O_2_ saturation, at all points trained men and untrained men significantly different than women. (bsln 1 and 2, Baseline 1 and 2; LBF_OCC_ 1 and 2, leg blood flow occlusion 1 and 2; deoxy[Hb+Mb]_PLATEAU_, deoxygenated hemoglobin concentration plateau). #, significantly different than all other time points; +, significantly different than bsln 1 & 2; ^, significantly different than LBF_OCC_ 1; †, significantly different than LBF_OCC_ 2; *, significantly different than bsln 1; ᶲ, significantly different than bsln 2.

Absolute change in deoxy[Hb+Mb], total[Hb+Mb], and tissue O_2_ saturation was evaluated throughout the course of the protocol (Fig 2A, 2B and 2C, respectively). deoxy[Hb+Mb] at all points in trained men were different than women (*p* < 0.05). total[Hb+Mb] for trained men were greater than untrained men at all time points (*p* < 0.05) whereas untrained men were not different than women, nor were differences found between trained men and women (*p* > 0.05). At each time point women displayed greater values of tissue O_2_ saturation compared to both trained and untrained men (*p* < 0.05).

### Deoxy[Hb+Mb] amplitude and $\dot{V}$O_2peak_

**Fig 3:**
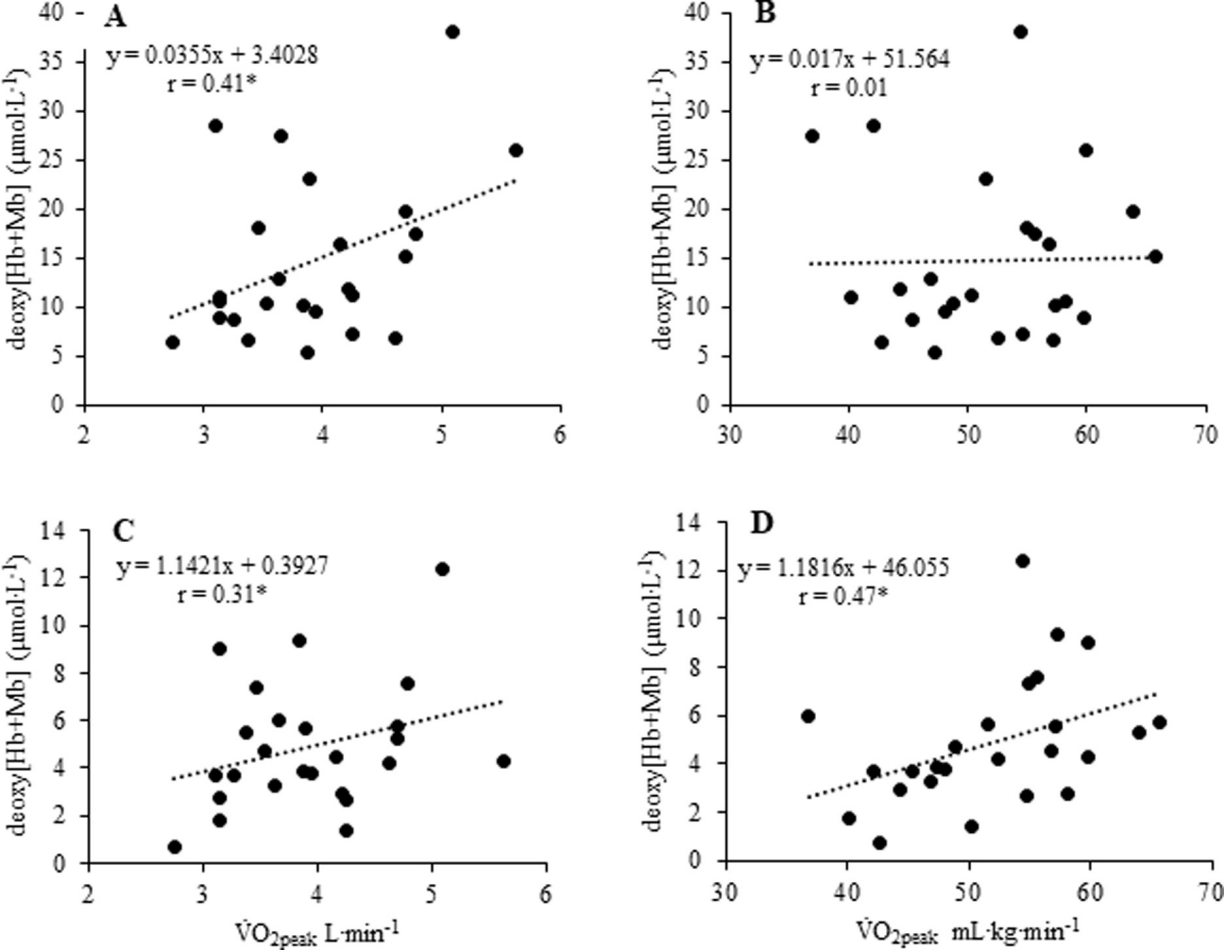
Relationship of the amplitude of NIRS-derived deoxyhemoglobin concentration (deoxy[Hb+Mb]) (μmol∙L^-1^) signal with $\dot{V}$O_2peak_ (absolute and relative values). *PANEL A)* Absolute $\dot{V}$O_2peak_ vs amplitude from bsln to deoxy[Hb+Mb]_PLATEAU_; *PANEL B)* Relative $\dot{V}$O_2peak_ vs amplitude from bsln to deoxy[Hb+Mb]_PLATEAU_; *PANEL C)* Absolute $\dot{V}$O_2peak_ vs amplitude from deoxy[Hb+Mb]_PLATEAU_ to LBF_OCC_ 2; *PANEL D)* Relative $\dot{V}$O_2peak_ vs amplitude from deoxy[Hb+Mb]_PLATEAU_ to LBF_OCC_ 2. (*, significant correlation, p < 0.05).

When pooled data were considered, a positive correlation was found between the amplitude of the deoxy[Hb+Mb] signal during the RI test and absolute (Fig 3A) but not relative $\dot{V}$O_2peak_ values (Fig 3B). A positive correlation was also found between the amplitude of the deoxy[Hb+Mb] signal reserve and both absolute and relative $\dot{V}$O_2peak_ values (*p* < 0.05) (Fig 3C and 3D).

### Deoxy[Hb+Mb] slope of increase during RI test

When normalized for percentage PO during the RI test, no difference was found between trained men (1.38 ± 0.36%∙s^-1^), untrained men (1.28 ± 0.32%∙s^-1^), and trained women (1.29 ± 0.16%∙s^-1^) for slope of the deoxy[Hb+Mb] signal during the RI until the plateau of the response (*p* > 0.05).

## Discussion

The present study investigated whether the magnitude of the O_2_ extraction reserve following a blood flow occlusion immediately at the end of ramp-incremental exercise was different in trained compared to untrained men and trained women. Our findings demonstrated that the observed O_2_ extraction reserve is present in groups differing in sex and training level, however, in contrast to our hypothesis we found that the magnitude of this reserve was not different amongst these groups.

### O_2_ extraction reserve

A novel finding of this study is the existence of an O_2_ extraction reserve in untrained and trained men as well as in trained women, which is in line with recent evidence indicating that the deoxy[Hb+Mb]_PLATEAU_ that is generally observed towards the end of a RI test to exhaustion [3,22,27,31–33] does not represent the upper limit in O_2_ extraction [3,4]. The presence/magnitude of the O_2_ extraction reserve may be mainly related to the presence of a reserve of oxygenated hemoglobin in the area of NIRS interrogation [3]. It is important to consider, however, that due to the overlapping of the Hb and Mb spectra within the near-infrared light range, the NIRS does not distinguish their relative impact to the various signals [8]. Thus, given that full desaturation of Mb is achievable only under ischemic conditions [34], and that there are contrasting views on the relative contribution of Hb and Mb to the NIRS signals [8,35], it cannot be excluded that some O_2_ bound to myoglobin, although small, contributed to the magnitude of the reserve.

Based on the concept that $\dot{V}$O_2_ is the product of blood flow and oxygen extraction, a plateau in O_2_ extraction would imply a greater perfusive supply in the presence of an increasing $\dot{V}$O_2_. This idea is supported by the fact that the onset of the deoxy[Hb+Mb]_PLATEAU_ is associated with other exercise intensity landmarks (i.e., RCP, maximal lactate steady-state, and critical power) [31,36–39], above which there is a progressive accumulation of metabolites (e.g., ATP, K^+^, [BLa], H^+^) that are known to promote local vasodilatory responses [40]. The current study supports this coincidence as no differences were found between the $\dot{V}$O_2_ associated with the RCP and the deoxy[Hb+Mb]_BP_ in any of the groups. Although the total[Hb+Mb] signal is not a proxy of blood flow, it is important to consider that the total[Hb+Mb] increased from baseline to the end of the ramp incremental test in trained and untrained men but this was not the case in women in which the total[Hb+Mb] was not statistically higher at the deoxy[Hb+Mb]_PLATEAU_.

It could be argued that the plateau in the deoxy[Hb+Mb] signal is simply representative of the finite O_2_ diffusive capacity of the muscle at near maximal exercise intensities [41,42], and that the O_2_ extraction reserve found immediately after ramp-exercise results from “unutilized” O_2_ that was bound to capillary Hb draining from active and non/or less active muscle fibers. While we do not neglect the possibility that an O_2_ diffusive limitation may partly contribute to the manifestation of the deoxy[Hb+Mb]_PLATEAU_, it is important to note that this plateau occurs at approximately 80% of $\dot{V}$O_2peak_. Hypothesizing a diffusive limitation of O_2_ without any increase in muscle perfusion implies that from the intensity associated to the onset of the deoxy[Hb+Mb]_PLATEAU_ up to the end of exercise (i.e., $\dot{V}$O_2peak_ at the level of the mouth), muscle $\dot{V}$O_2_ in the area of the VL muscle did not increase further. Although other muscle areas in the exercising limbs as well as the greater work of the respiratory muscles contribute to this further increase in the $\dot{V}$O_2_ expressed at the level of the mouth (i.e., from 80% of $\dot{V}$O_2peak_), it is unlikely that these muscle areas alone can account for the remaining increase in $\dot{V}$O_2_ [3], especially considering the continued (and disproportionate) activation of the VL muscle at this exercise intensity [43]. Therefore, we reason that vascular dynamic control (that may arise from the interplay of fiber type expression and differences in patterns of muscle activation) may favor the redistribution of blood from muscle areas that are less metabolically challenged to those of greater demand.

### O_2_ extraction reserve: Trained vs untrained and men vs women

Greater systemic arteriovenous O_2_ difference [20] as well as the larger amplitude of the deoxy[Hb+Mb] signal measured during ramp-exercise [21] in trained compared to untrained individuals indicate a relationship between $\dot{V}$O_2peak_ and muscle O_2_ flux [44]. Based on this premise, we hypothesized that the muscle of trained individuals would be able to extract more O_2_ from the surrounding microvasculature, thus leading to a reduced or eliminated O_2_ extraction reserve in comparison to untrained counterparts. Although we found a correlation (though moderate) between $\dot{V}$O_2peak_ and the amplitude of the deoxy[Hb+Mb] signal during ramp-exercise, contrary to this hypothesis, we did not find any difference in the magnitude of the reserve between trained and untrained individuals. The fact that we were unable to detect any difference across groups can be explained by different reasons. First, it is important to acknowledge that despite the difference in aerobic fitness, the slope of the increase in the deoxy[Hb+Mb] signal during ramp-exercise was similar across the groups investigated, which may indicate that the rate of O_2_ diffusion in the present study was independent of training levels. Second, it could be possible that the magnitude of the O_2_ extraction reserve may not only be dependent on the individual maximal O_2_ extraction capacity, but also on the amount of oxygenated blood perfusing the area investigated immediately prior to the blood flow occlusion [4]. In this perspective, exercise training adaptations improve the matching of local blood flow with O_2_ utilization as well as reduce sympathetically-induced vasoconstriction during exercise [45]. Thus, trained individuals may not necessarily demonstrate a smaller O_2_ extraction reserve simply based on a greater oxidative capacity.

Although it was hypothesized that the greater reliance by women on fractional O_2_ extraction for a given increase in $\dot{V}$O_2_ (due to a lower efficiency in redistributing blood flow to the active tissues and/or lower hemoglobin carrying capacity [22,27]), would result in a smaller O_2_ extraction reserve compared to men, the present data did not support this idea, as no differences in magnitude of the reserve were observed between trained or untrained men and trained women. In this regard, however, it is important to note that there exist divergent findings on the hemodynamics responses to exercise of women compared to men. Indeed, although Murias et al. [22] hypothesized a poorer matching between blood flow and O_2_ utilization in women compared to men at higher intensities of exercise (i.e., above ~50% of $\dot{V}$O_2peak_), other studies have shown that women may have a better vasodilatory reactivity that may originate from an attenuated α-adrenergic sensitivity [46] and other physiological mechanisms that remain elusive at the moment [47]. However, it needs to be acknowledged that these two studies evaluated vasodilatory reactivity at rest [46] or during submaximal forearm exercise [47].

### Peak deoxy[Hb+Mb] signal during occlusion at “rest” vs at $\dot{V}$O_2peak_

Some studies have performed blood flow occlusions of the investigated limb either at rest or a few minutes after an exercise protocol to “physiologically calibrate” the NIRS device [7,9,48–51], with the overall amplitude of the deoxy[Hb+Mb] signal during the transient ischemia supposedly representing the range of O_2_ extraction from rest to complete desaturation [52]. Generally with this procedure the peak deoxy[Hb+Mb] values at maximal exercise are found to be approximately 60–70% of the values achieved in the blood-flow occlusion that is performed during a recovery period following maximal exercise [53–55]. The interpretation of these findings would be that the O_2_ extraction reserve can be assessed by simply performing the occlusion at rest or a few minutes after exercise when the $\dot{V}$O_2_ response is close to baseline values, with the implicit assumption that the amplitude of the deoxy[Hb+Mb] signal is “insensitive” to differences in metabolic rate (e.g., rest vs $\dot{V}$O_2peak_) and volume-related factors (such as microvascular hematocrit and/or capillary recruitment). However, the present study found that there were no differences between the amplitudes achieved during the RI test (i.e., deoxy[Hb+Mb]_PLATEAU_) and those achieved during the LBF_OCC_ 1 (i.e., resting condition), with the highest amplitudes in the deoxy[Hb+Mb] signal (~30–40% greater) found during the LBF_OCC_ 2 (i.e. immediately upon termination of the RI test).

Although these contrasting findings are difficult to reconcile, it is important to note that the amplitude of the deoxy[Hb+Mb] signal during an occlusion is likely to be affected, as previously mentioned, by the metabolic rate and the volume of blood in the area of interrogation. Indeed, resting metabolic conditions prior to the RI test may not necessarily be sufficient to induce the highest possible level of extraction compared to at $\dot{V}$O_2peak_ (i.e., highest metabolic rate and corresponding perfusion affecting O_2_ tension). Furthermore, the volume of blood may be a contributing factor, as the deoxy[Hb+Mb] signal may not be independent from changes related to hematocrit [56]. From this perspective, when the occlusion is performed “a few minutes” after ramp-exercise the amplitude of the deoxy[Hb+Mb] signal may be affected by the hyperemic response during recovery. Therefore, an important difference is that compared to the current study, none of the previously mentioned studies performed a blood-flow occlusion immediately upon exercise termination.

### Methodological considerations

While the results of this study indicate no significant differences between men and women in the amplitude of the reserve, it could also be interpreted as the inability of the NIRS-derived deoxy[Hb+Mb] signal to differentiate between groups due to the variability in the amplitude of the signal. Even after “correcting” for the adipose tissue thickness to mitigate the effect of subcutaneous fat on the NIRS signal, inter-individual differences in terms of amplitude are rather large and these dissimilarities greatly affect the associated relative change and increase the variability of the data [52]. Therefore, the comparison between groups becomes problematic and the likelihood of finding significant differences is reduced. In this perspective, the present study did not evaluate the O_2_ extraction response in untrained women due to a generally higher amount of subcutaneous fat underneath the area of NIRS probe interrogation resulting in a minimal amount of amplitude in the NIRS signals. Despite the lack of data on untrained women, however, it is important to incorporate the results from the trained women into the scientific literature as less is known about the responses in women. Furthermore, although the study was sufficiently powered (>0.8) to detect statistical difference in the level of O_2_ extraction at different time points, the reader should be aware that the relatively low sample size may have prevented us from detecting between group differences in the deoxy[Hb+Mb] reserve as well as in the total[Hb+Mb] signal, thus the results should be interpreted with caution.

In addition, it is known that a period of blood flow occlusion would result in hypoxic conditions within the muscle and increase the accumulation of metabolites, which may impact on the profile of the O_2_ extraction. However, given that the peak of the O_2_ extraction reserve immediately after exercise is shown within a short period of time after occlusion and maintained throughout its duration, it is unlikely that the further metabolic perturbations affected the magnitude of the reserve.

Finally, although the present data suggest that improved perfusion within the area of NIRS interrogation is responsible for the plateau in the deoxy[Hb+Mb] signal observed towards the end of the RI test, and that the further increase in the deoxy[Hb+Mb] signal after occlusion further supports this contention, it could be argued that the O_2_ extraction reserve could be attributed to blood volume shifts (i.e., redistribution of blood flow) associated with the cuff occlusion. It should be noted that the NIRS signal detects changes in the muscle under the area of probe interrogation, and thus the signal may not account for the heterogeneous responses that may be present within the same muscle at different sites and/or depths [32,57]. However, despite these potential heterogeneities, we are confident that the reserve is present throughout the VL muscle as well as in other muscles of the quadriceps, as the deoxy[Hb+Mb] reserve has been identified in both superficial and deep portions of the vastus lateralis as well as in the rectus femoris [4].

## Conclusions

The results of this study demonstrated the existence of an O_2_ extraction reserve that was consistently observed in all participants. In contrast to our hypothesis, there was no sex- (trained men vs trained women) or training status-related (trained men vs untrained men) influence on the amplitude of the O_2_ extraction reserve, despite the potential for differences to exist. Collectively, these data support the idea that in the presence of a levelling off in the O_2_ extraction signal in the VL muscle, a further increase in $\dot{V}$O_2_ within this region might be supported by an increase in local provision of O_2_ irrespective of sex- or training-related differences.
